# Shape and Size-Dependent Magnetic Properties of Fe_3_O_4_ Nanoparticles Synthesized Using Piperidine

**DOI:** 10.1186/s11671-017-2039-3

**Published:** 2017-04-26

**Authors:** Ashwani Kumar Singh, O. N. Srivastava, Kedar Singh

**Affiliations:** 1 School of Physical Sciences, Jawaharlal Nehru University, New Delhi, 110067 India; 2 Department of Physics, Banaras Hindu University, Varanasi, 221005 India

## Abstract

In this article, we proposed a facile one-step synthesis of Fe_3_O_4_ nanoparticles of different shapes and sizes by co-precipitation of FeCl_2_ with piperidine. A careful investigation of TEM micrographs shows that the shape and size of nanoparticles can be tuned by varying the molarity of piperidine. XRD patterns match the standard phase of the spinal structure of Fe_3_O_4_ which confirms the formation of Fe_3_O_4_ nanoparticles. Transmission electron microscopy reveals that molar concentration of FeCl_2_ solution plays a significant role in determining the shape and size of Fe_3_O_4_ nanoparticles. Changes in the shape and sizes of Fe_3_O_4_ nanoparticles which are influenced by the molar concentration of FeCl_2_ can easily be explained with the help of surface free energy minimization principle. Further, to study the magnetic behavior of synthesized Fe_3_O_4_ nanoparticles, magnetization vs. magnetic field (M-H) and magnetization vs. temperature (M-T) measurements were carried out by using Physical Property Measurement System (PPMS). These results show systematic changes in various magnetic parameters like remanent magnetization (Mr), saturation magnetization (Ms), coercivity (Hc), and blocking temperature (*T*_B_) with shapes and sizes of Fe_3_O_4_. These variations of magnetic properties of different shaped Fe_3_O_4_ nanoparticles can be explained with surface effect and finite size effect.

## Background

Nano-sized materials on account of their surface and quantum size effect not only are known to possess better physical and chemical properties but also have enhanced biocompatibility and bioefficacy [1, 2]. In this context, magnetic nanoparticles for their unique magnetic behavior have gained much attention in recent years, whereby they are known to have promising potential for various medical applications such as targeted drug delivery systems, MRI, diagnostics, radiofrequency hyperthermia, and cancer therapy [3–7]. Besides, magnetic nanoparticles are also being utilized as a key material for magnetic ferrofluid [8], catalysis [9], data storage [10], and environmental remediation [11]. Fe_3_O_4_, a magnetic nanoparticle, has the cubic inverse spinal structure (two Fe^3+^ with one Fe^2+^) in which oxygen forms an fcc closed-pack structure [12]. It is an important class of half-metallic materials, as electrons hop between Fe^2+^ and Fe^3+^. However, their utilization for practical application still requires rectification of several parameters, broadly categorized into two main class: (a) their tendency to get aggregate in order to reduce their surface energy and (b) their ability to get oxidize easily. The aforementioned parameters can hamper their interfacial area, thereby hindering their magnetism and dispersibility. Henceforth, it becomes essentially important to overcome such parameters which possibly can be achieved by developing potential synthesis methods which overrule such problems. With the advent of several wet chemical methods for the synthesis of nanoparticles in the recent past, the magnetic nanoparticles have been synthesized by different methods such as solvothermal [13], sol-gel [14], co-precipitation [15], thermal decomposition [16], and sonochemical reaction [17]. Here in this work, we have designed a new and facile one-step synthesis of Fe_3_O_4_ nanoparticles by using a new chemical piperidine (C_5_H_11_N) by hydrolysis method. Amongst several chemicals such as ether (CH_3_OCH_3_) and formaldehyde (HCHO), piperidine was found most effective for the synthesis of Fe_3_O_4_ nanoparticles.

## Experimental

*Chemicals*: FeCl_2_·4H_2_O (anhydrous) was procured from Sigma-Aldrich, while piperidine (C_6_H_5_N) was procured from Merck. All chemicals were used as received. Double-distilled water was used in reaction as a medium.

## Preparation of Fe_3_O_4_ Nanoparticles

Synthesis of Fe_3_O_4_ nanoparticles was made in four different sets (by varying the molarity of FeCl_2_ solution) to study the influence of the reaction parameters on the size and shape of Fe_3_O_4_ nanoparticles. A solution of piperidine (50 ml, 0.25 M) was prepared by mixing 1.24 ml piperidine (C_5_H_11_N) homogeneously into 50 ml double-distilled water. This was used as stock solution throughout the experiments. The solutions of FeCl_2_ (10 ml) with varying molarity (0.025, 0.05, 0.075, and 0.1 M) was prepared by dissolving 0.0497, 0.0994, 0.1491, and 0.1988 g FeCl_2_ in double-distilled water, respectively. These samples were designated as S_1_, S_2_, S_3_, and S_4_, respectively. Now, 5 ml of prepared piperidine solution was mixed with FeCl_2_ solution of different molarities as in above, under stirring. An instant change in color indicated the formation of Fe_3_O_4_ nanoparticles. The reaction mixture was then centrifuged at 10,000 rpm for 10 min. Particles were collected and resuspended in 5 ml double-distilled water for further characterizations.

## Characterization

The XRD analyses of resulting samples were carried out with an X-Pert Pro X-ray diffractometer (PAN analyst BV the Netherlands with a build in graphite monochromator meter) with Cu Kα radiation (*λ* = 1.54056 A°). Sample preparation for XRD was done by placing one drop of the reaction mixture on a circular disk (5 mm diameter) and allowing it to dry. Transmission electron microscopic (TEM) studies were done by employing TECHNI 20 G^2^ microscope at an accelerating voltage 200 KeV. Samples for TEM were prepared by suspending powder in double-distilled water and ultrasonicated it for 1 h. The suspension obtained was placed on a formvar-coated Cu grid. Magnetic measurements were performed on 14 T Physical Properties Measurement System, Cryogenics Limited, USA.

## Discussion

**Fig. 1:**
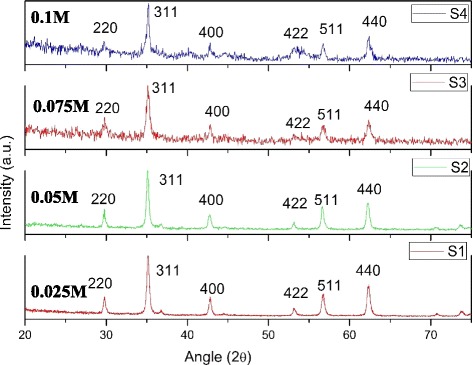
The XRD profile of Fe_3_O_4_ nanoparticles synthesized with different concentrations of FeCl_2_ solution

Structural and microstructural characterization of the samples were investigated by using XRD pattern. Figure 1 represents the XRD profile of Fe_3_O_4_ nanoparticles synthesized with different concentrations of FeCl_2_ solution.

The XRD pattern can be matched to the series of Bragg reflections corresponding to the standard phase of the spinal structure of Fe_3_O_4_ with a lattice constant of *a* = 8.41A° (ICSD82-1533). Six peaks at 30.16°, 35.49°, 43.01°, 53.78°, 57.21°, and 62.73° can be indexed as (220), (311), (400), (422), (511), and (440) of the cubic structure (Fd3m space group) of Fe_3_O_4_ nanoparticles.

**Fig. 2:**
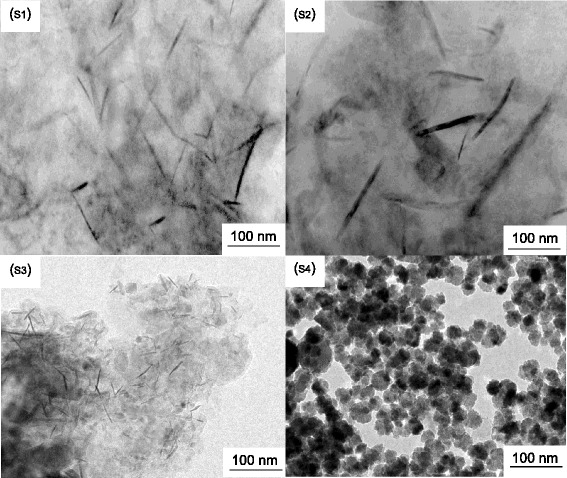
TEM micrographs of as-synthesized Fe_3_O_4_ with varying molarity of FeCl_2_. Notice the change in morphology with varying molarity

Intensive TEM analysis was performed to investigate the shape and size of the as-synthesized Fe_3_O_4_ particles using piperidine. Figure 2 (S_1_–S_4_) depicts the typical TEM micrographs of Fe_3_O_4_ particles synthesized with varying molarity of FeCl_2_ (0.025, 0.05, 0.075, and 0.1 M) with respect to piperidine. Figure 2 (S_1_) depicts the TEM micrograph of the synthesized Fe_3_O_4_ particles synthesized with a least molar concentration of FeCl_2_ (0.025 M). As it can be seen from Fig. 2 (S_1_), particles are nearly rod-shaped with high aspect ratio (~10) and having a length between 150 and 200 nm. As it is clearly visible that rods are tapered at the ends and maximum at the center, making it a needle-like structure, from Fig. 2 (S_2_–S_4_), it can be seen that with increasing molarity of FeCl_2_ solution, aspect ratio of rod-like nanostructures decreases. At the highest molarity of FeCl_2_ solution (0.1 M), Fe_3_O_4_ nanoparticles become spherical in morphology. As we know that rate of reaction plays a dominant role in the shape and size of nanocrystals, during the process of formation of nanocrystals, the growth rate is different for different crystallographic planes which are based on surface free energy minimization principle. Further, the ratio of the growth rate of different directions determines the shape of the crystal [18]. Based on the above theory, the facets with higher energy grow faster and tend to disappear, which leads to the crystal to bind by low-energy facets. This results in different morphologies of Fe_3_O_4_ crystals. In solution phase synthesis, it is well known that capping agents can change the free energy of different facets through their interaction with crystal surface [19, 20]. In our synthesis protocol, piperidine is used which acts as both reductant and surfactant. During the reaction of FeCl_2_ with piperidine, somewhere in intermediate stage Fe(OH)_2_ is formed. As the molar concentration of FeCl_2_ is in increasing order from S1 (0.025 M) to S4 (0.1 M), formation of Fe(OH)_2_ complex sharply increases with increasing concentration of FeCl_2_. This fast formation rate consequence in merging the nucleation and growth steps. A separate nucleation and growth steps are a major factor for the high-quality anisotropic growth of the crystal. Therefore, a lower molar concentration of FeCl_2_ anisotropic (rod-shaped) growth of nanoparticles is observed. As we increase the concentration of FeCl_2_ isotropic growth, it replaces the directed growth of Fe_3_O_4_ crystals due to the high concentration of FeCl_2_. Since nucleation and growth steps are not separate in case of high-concentration samples, these nucleated nanocrystals of Fe_3_O_4_ tend to form Fe_3_O_4_ sphere without any specific growth directions, which is thermodynamically favored morphology.

### Magnetic Properties of Nano-sized Fe_3_O_4_

**Fig. 3:**
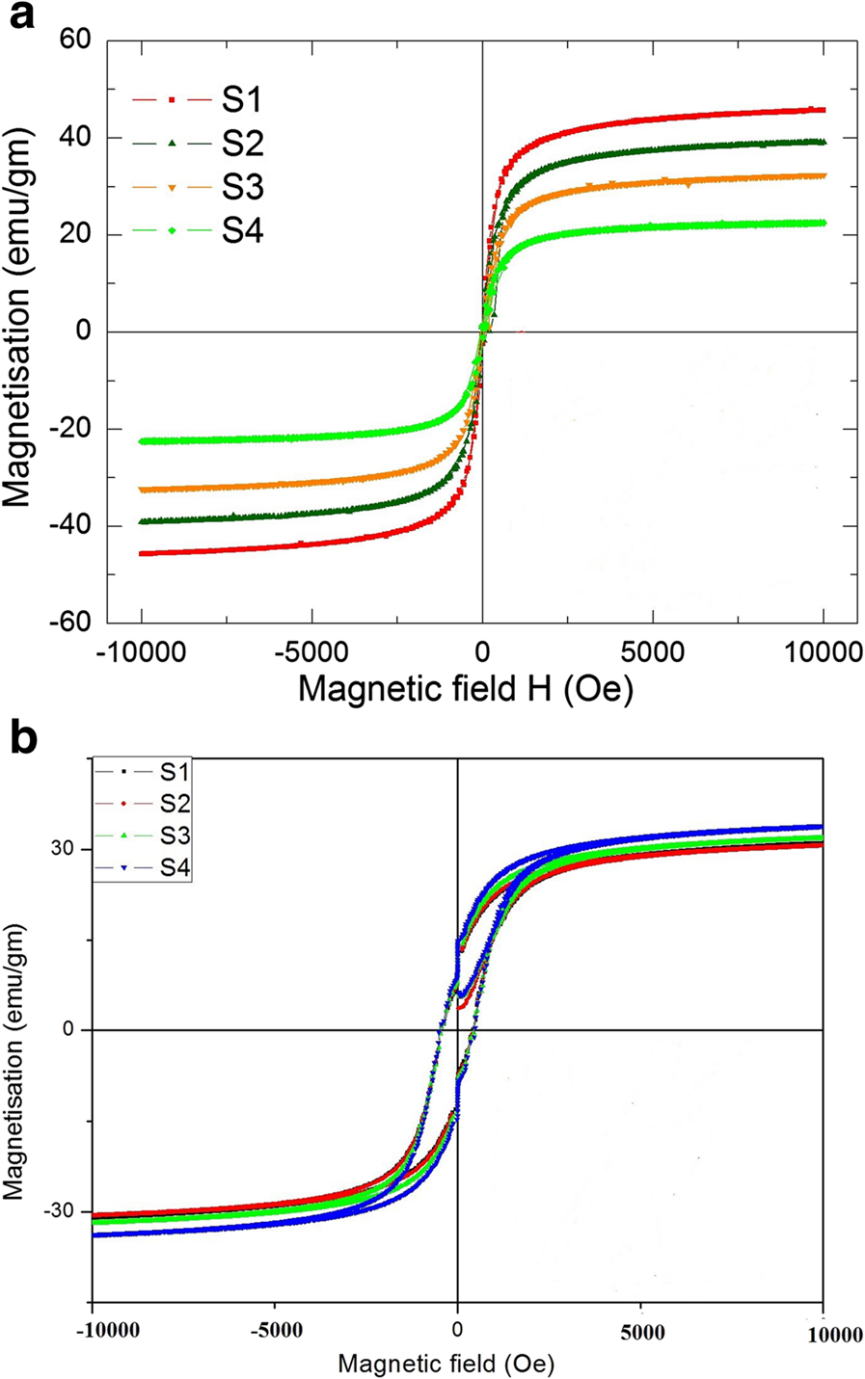
**a** Magnetization vs. magnetic field plot of different Fe_3_O_4_ samples at 300 K. **b** Magnetization vs. magnetic field plot of different Fe_3_O_4_ samples at 5 K

**Fig. 4:**
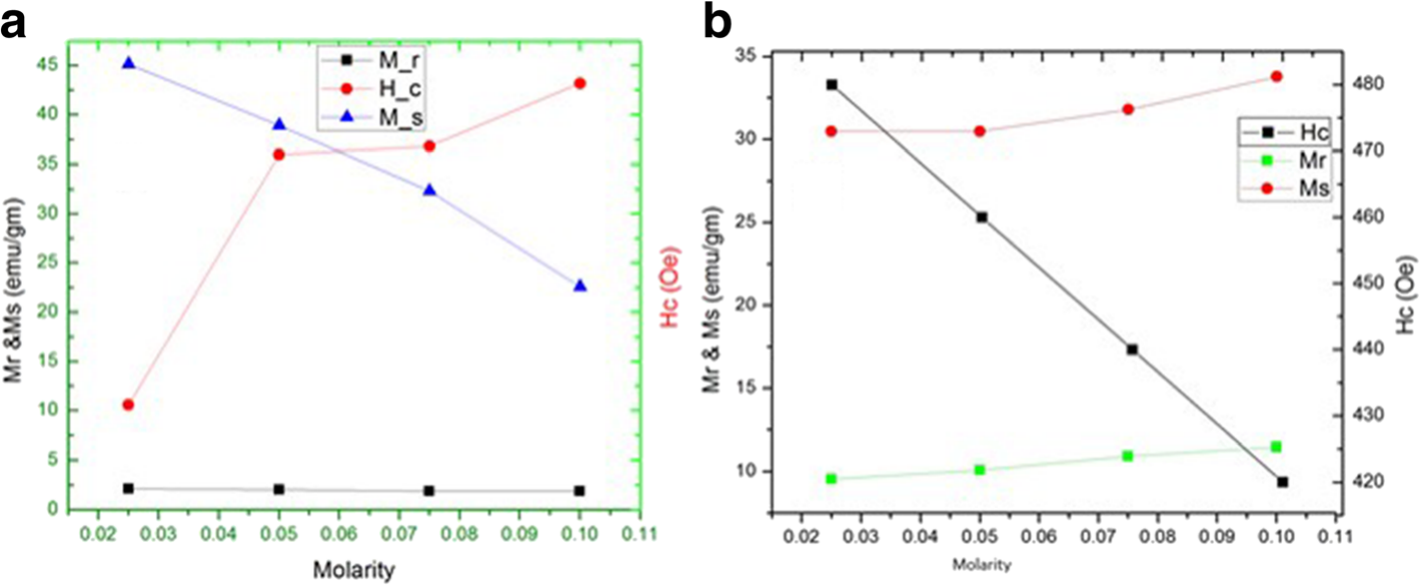
**a**, **b** Variation of Mr, Ms and Hc as a function of molarity for different samples at 300K and 5K respectivly

The magnetization vs. magnetic field (M-H) variations of different shaped and sized Fe_3_O_4_ samples were analyzed by using Physical Properties Measurement System (PPMS) having the facility to vary the magnetic field up to 14 T. Hysteresis loops of various Fe_3_O_4_ samples have been shown in Fig. 3a, b recorded at 300 and 5 K, respectively. These figures give some useful information about the magnetic response of various samples. All samples show a nonlinear variation in magnetization as a function of magnetic field at both temperatures (300 and 5 K). Figure 3a is a typical M-H curve for all four samples at measured at 300 K. It is evident that magnetic remanence is almost absent in all samples. Another peculiar feature is initial slopes in magnetization curves at 300 K of all samples are very steep. These observations can be explained by surface effect and finite size effect. Incorporation of these effects on a magnetic system suggests that particles are small enough to be considered as single-domain particles. These single domains orient as a large single magnetic moment in the direction of applied field. Because of the single-domain nature of particles, it shows almost no remnant magnetization after removal of external applied magnetic field. The values of Mr, Ms, and Hc of all samples are given in the table. Further, Fig. 3b shows the M-H plot of the same samples at 5 K. The behavior of this graph is very different from the M-H plot at 300 K. The distinct feature of this graph is Hc which began to appear with large value with respect to Hc at 300 K. This results in the disappearing superparamagnetic behavior of particles. Further in this case (Fig. 3b), saturation magnetization is almost the same for all samples, but in Fig. 3a, different samples show different saturation magnetization. Figure 4a, b is the plot of Mr, Ms, and Hc as a function of molarity calculated from inset of Fig. 3a, b, at 300 and 5 K, respectively. A careful observation of Fig. 4a indicates that the coercivity (Hc) of the samples have been found to increase monotonously with the decrease in the aspect ratio of the magnetic nanostructures (from 10.60 to 42.30 Oe). The Hc value of the rod-shaped nanostructures having the largest aspect ratio (S_1_) is the least and vice versa. It indicates that the magnetization of the elongated particles is more sensitive to the applied field than that of the particles having less aspect ratio [22]. These figures show explicitly the effect of temperature on Mr, Ms, and Hc. A notable change appears in Hc as we cool the samples up to 5 K. Hc monotonously decreases for all samples as we increase the temperature. This behavior can be understood as due to enhancement in thermal energy via temperature will enhance the thermal fluctuations of pinned magnetic moments, therefore, minimizing the effect of anisotropy barriers. This appearance of coercivity at low temperature destroys the superparamagnetic behavior of Fe_3_O_4_ nanoparticles, which is the characteristic feature of Fe_3_O_4_ nanoparticles at 300 K. Numerical values of Mr, Ms, and Hc have also been incorporated in the table. The data recorded at 300 K are much improved than reported earlier [21].

**Fig. 5:**
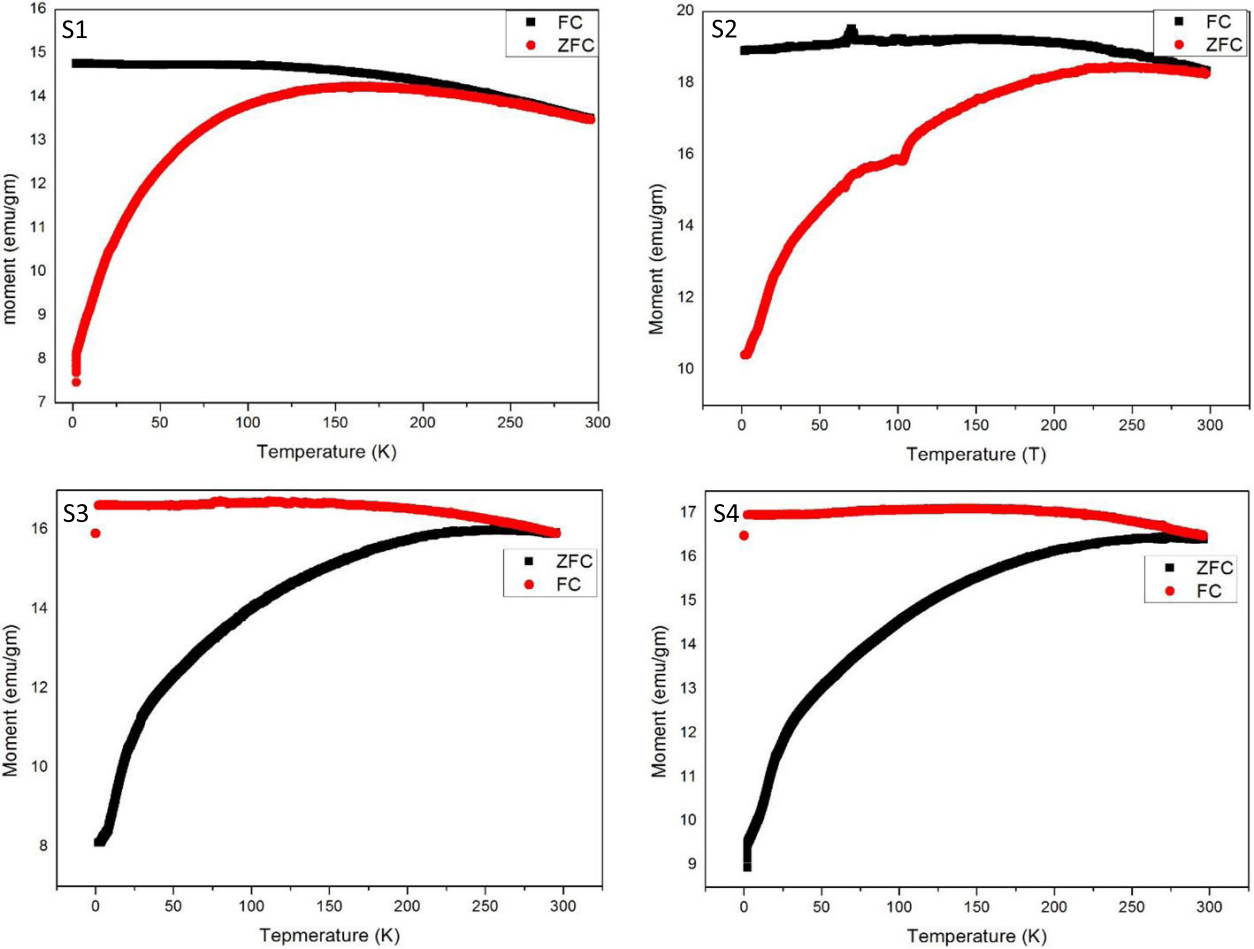
Magnetisation vs temperature plot of different Fe_3_O_4_ ­samples (S1-S4)

Further, magnetization vs. temperature (M-T) measurement of as-prepared Fe_3_O_4_ nanoparticles of different shapes and sized was investigated using PPMS. Zero-field-cooling (ZFC) and field-cooling (FC) processes were used to study the magnetization vs. temperature profile of synthesized nanoparticles between 5 and 300 K at 500 Oe (Fig. 5). FC and ZFC curves are usually utilized to understand the energy barriers [23]. Figure 5 shows the magnetization response as a function of temperature for all four samples (S_1_, S_2_, S_3_, and S_4_). All samples were in powder form while undergoing for measurement. When the sample is cooled at very low temperature (~5 K), the net magnetic moment is negligibly small as the magnetic moment of every individual particle is randomly oriented. When the external magnetic field is applied, randomly oriented moments begin to align in the direction of the field. Therefore, net magnetic moment increases gradually and reaches up to a maximum (169, 246, 250, and 266 K for S_1_, S_2_, S_3_, and S_4_, respectively). The temperature at which magnetization is maximum is known as blocking temperature (*T*_B_). This is also defined as the temperature at which thermal energy is in equilibrium with the energy of aligned magnetic moments. As the temperature rises greater than the blocking temperature, thermal energy begins to destroy the alignment of moments and hence resulting in a decline of magnetization above *T*_B_. Further, it may be pointed out from graphs that FC and ZFC curves behave in a similar way above blocking temperature which is different for all samples [24, 25].

Some useful parameters of M-T and M-H measurements have been given in Table 1.

**Table 1:** Some useful parameters of M-T and M-H measurements

Sample name	Mr (emu/g) at 300 K	Ms (emu/g) at 300 K	Hc (Oe) at 300 K	Mr (emu/g) at 5 K	Ms (emu/g) at 5 K	Hc (Oe) at 5 K	*T*_B_ (K)
S_1_	2.13	45.15	10.60	9.54	30.49	480	169
S_2_	2.01	38.95	36.00	10.17	30.44	465	246
S_3_	1.90	32.30	36.85	10.91	31.81	440	250
S_4_	1.87	22.60	43.20	11.45	33.78	425	266

### Mechanism of Formation of Fe_3_O_4_ Nanoparticles

Our synthesis protocol initially requires the aqueous solution of FeCl_2_. Reaction is as follows:

$$ {\mathrm{FeCl}}_2+2{\mathrm{H}}_2\mathrm{O}\kern1.08em \to \kern1.08em \mathrm{F}\mathrm{e}{\left(\mathrm{OH}\right)}_2+2\mathrm{H}\mathrm{C}\mathrm{l} $$

**Fig. 6:**
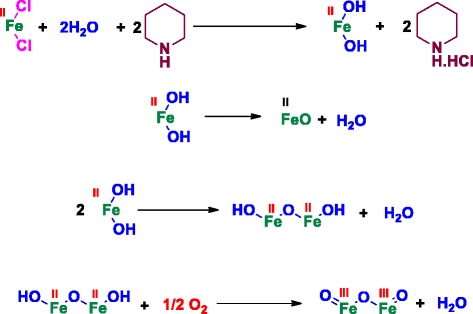
(1) The first step is the hydrolysis of FeCl_2_ by water in presence of piperidine which facilitates the hydrolysis by neutralizing HCl produced in the reaction and hence making the reaction move in forward direction. (2) The next step is dehydration of Fe(OH)_2_ to produce ferrous oxide. (3) The similar dehydration of two molecules of ferrous hydroxide produces H_2_Fe_2_O_3_ which on spontaneous aerial oxidation produces the Fe_2_O_3_. (4) The FeO and Fe_2_O_3_ combine to give Fe_2_O_4_

This is a reversible reaction with almost equal forward and backward reaction rate. After adding piperidine in the reaction mixture, HCl gets trapped with piperidine, making the reaction in the forward direction only. This leads to the formation of stable Fe(OH)_2_. This Fe(OH)_2_ undergoes dehydration process to produce FeO. Similar process occurs with two FeO molecules resulting in the formation of H_2_Fe_2_O_3_, which on spontaneous aerial oxidation gives Fe_2_O_3_. In the final step of the reaction, FeO and Fe_2_O_3_ get combined to produce Fe_3_O_4_. This whole process is represented in pictorial form (Fig. 6).

## Conclusion

Conclusions of the current study can be summarized as follows:

1. Fe_3_O_4_ nanoparticles are indeed synthesized using piperidine, which is confirmed by XRD characterization of as-synthesized samples.
2. TEM images give some useful information related to the shape and sizes of the particles. Our investigation shows that shape and size of the particles can be changed from rods to spheres by varying the molar concentration of FeCl_2_ solutions (from 0.025 to 0.1 M).
3. Measurement of magnetic properties found after deep analysis shows that these magnetic parameters like Ms, Mr, Hc, and *T*_B_ have shown improved values than reported earlier.
4. These synthesized Fe_3_O_4_ nanoparticles of different shapes and sizes will further be used for their applications like EMI shielding. Some primitive experiments are going on and very soon will be followed by respective publications.
